# Game Theoretic Clustering for Finding Strong Communities

**DOI:** 10.3390/e26030268

**Published:** 2024-03-18

**Authors:** Chao Zhao, Ali Al-Bashabsheh, Chung Chan

**Affiliations:** 1 Department of Computer Science, City University of Hong Kong, Hong Kong, China; ao.ao@my.cityu.edu.hk; 2 School of General Engineering, Beihang University, Beijing 100191, China; entropyali@gmail.com

**Keywords:** game theory, community detection, hierarchical clustering

## Abstract

We address the challenge of identifying meaningful communities by proposing a model based on convex game theory and a measure of community strength. Many existing community detection methods fail to provide unique solutions, and it remains unclear how the solutions depend on initial conditions. Our approach identifies strong communities with a hierarchical structure, visualizable as a dendrogram, and computable in polynomial time using submodular function minimization. This framework extends beyond graphs to hypergraphs or even polymatroids. In the case when the model is graphical, a more efficient algorithm based on the max-flow min-cut algorithm can be devised. Though not achieving near-linear time complexity, the pursuit of practical algorithms is an intriguing avenue for future research. Our work serves as the foundation, offering an analytical framework that yields unique solutions with clear operational meaning for the communities identified.

## 1. Introduction

Community detection is a fundamental problem in various fields, such as biological study and social network analysis. The definition of a community can vary based on the specific problem and objective at hand, but the definitions provided in [1,2,3] are generally considered widely accepted. In broad terms, a community is commonly understood as a group of individuals with stronger connections among its members than with individuals outside the group.

In the process of conducting community detection, real-world problems are typically translated into graphs where nodes represent individuals and edges represent relations. Numerous community detection methods have been developed based on diverse principles and objective functions. Surveys of community detection methods can be found in [4,5,6,7,8,9,10].

Game theory has emerged as a technique applied in community detection [8,11,12,13,14,15]. Its applications extend to identifying disjoint, overlapping, and hierarchical communities. As a systematic framework, game theory models and studies the decisions and outcomes of players in a game [16,17]. Broadly, game theory can be categorized into two main types: non-cooperative game theory and cooperative game theory. Non-cooperative game theory focuses on the competition between individual players, emphasizing their strategies and payoffs. Cooperative game theory, on the other hand, focuses on the cooperation between players and addresses the allocation of payoffs to players based on the worth of the coalitions formed. Within cooperative game theory, there are two main types: non-transferable utility cooperative games, where the payoff for a player within a coalition cannot be transferred to another player in the same coalition, and transferable utility cooperative games, where payoffs are considered transferable among players in the same coalition. Solution concepts such as the core, kernel, nucleolus, Shapley value, egalitarian, etc., play crucial roles in cooperative game theory [18,19].

The community detection method based on cooperative game theory typically identifies the coalition with the highest score determined by a measure evaluated on the coalitions. However, due to the use of approximations, non-unique results are common. Zhou et al. [20] presented a community detection method using cooperative game theory and the Shapley value. The study focused on a social network where nodes are linked to relationships in various finite topics. The Shapley value represents a node’s contribution to the connection closeness of a coalition. The algorithm forms hierarchical and overlapping coalitions by iteratively adding each node to one of the coalitions formed in the previous iteration, where the newly added node obtains the largest Shapley value. Despite running in polynomial time, the algorithm relies on approximation. Another related approach for overlapping and hierarchical community detection [21] also employs cooperative game theory, and the hierarchical structure of coalitions is obtained through a greedy agglomerative method, potentially yielding non-unique results.

The Naming Game [22,23,24] presents another game theoretic approach applicable to community detection, where the community structure emerges from the dynamic interactions between pairs of nodes within the game. However, empirical evidence suggests that the solution is generally not unique [24]. The convergence and computational costs of the method are analyzed through extensive empirical experiments [22,24], while it remains unclear regarding the theoretical bounds. Furthermore, the Naming Game relies on pairwise connections and does not capture higher-order statistics among nodes beyond pairwise relationships, therefore limiting its scope of applications in community detection.

We introduce a notion of strength derived from cooperative game theory to identify strong communities that are interpretable. Moreover, the strong communities are unique, computable in polynomial time with recursive procedures, and can be represented by a dendrogram. The scope of consideration encompasses a set of individuals with a supermodular function for evaluating the communities, which means our approach is applicable to community detection tasks beyond graphical models. Our framework focuses on elucidating the theoretical properties of the strong communities and can provide the foundation for future research on empirical algorithms for large-scale datasets.

This paper is structured as follows: In Section 2, we present the relevant concepts in cooperative games. Section 3 outlines the derivation of the objective function based on convex games. We also formulate the definitions for community strength and strong communities in this section. Moving on to Section 4, we delve into the discussion of the properties of strong communities, laying the foundation for their computation. Section 5 details the solution to the problem through submodular function minimization and, in certain cases, introduces the use of the max-flow min-cut algorithm as a more efficient method in practice. In Section 6, concrete examples are provided to demonstrate the computation of strong communities and the representation of the dendrogram of these communities. Finally, in Section 7, we conclude our work.

## 2. Cooperative Game

A cooperative game [17] is characterized by (V,g), where

- *V* is a finite set of players with |V|≥2, and
- $g:2^{V}\to R$ is a set function called the characteristic function, where g(C) is the worth of the coalition C⊆V, assuming players in *C* cooperate to form such coalition.

Denote the payoff allocation for the players as a vector

$$r_{V}=\left(r_{1},r_{2},\ldots ,r_{\left|V\right|}\right)\in R^{\left|V\right|},$$

with $r_{i}$ being the *i*-th element in $r_{V}$ as the payoff allocated for *i*-th player.

The total payoff in the coalition C⊆V is denoted as

(1) $$\begin{array}{c} r\left(C\right):=\sum_{i\in C}r_{i}. \end{array}$$

Furthermore, when *g* is a supermodular function, the game is called convex [17]. In this case, for ∀B,C⊆V,

(2) $$\begin{array}{c} g(B)+g(C)\leq g(B\cup C)+g(B\cap C). \end{array}$$

Or equivalently, for ∀B⊆C⊆V,i∈V∖B,

(3) $$\begin{array}{c} g(B\cup \{i\})-g(B)\leq g(C\cup \{i\})-g(C), \end{array}$$

where both sides are the increases in worth when a player *i* is added to a coalition. (3) means that the increase in worth, when a player adds to a coalition, is equal or larger than that for a larger superset coalition, i.e., the marginal worth is non-diminishing for convex games. For simplicity, *g* is thought to be normalized, i.e., g(∅)=0.

As for the payoff allocation, the transferable utility is considered here, i.e., the payoffs can be transferred between players in the same coalition. The core [18] is one of the relevant solution concepts in cooperative games, which is about the feasible allocation of payoffs to players.

The core of a game (V,g) is defined as [17]:

(4) $$\begin{array}{c} \mathrm{Core}\left(V,g\right):=\{r_{V}\in R^{\left|V\right|}|r\left(V\right)=g\left(V\right),r\left(C\right)\geq g\left(C\right),\forall C\subseteq V\}. \end{array}$$

In the definition of the core, r(V)=g(V) means the payoff allocation exactly splits the total worth of the grand coalition *V*. The inequality r(C)≥g(C) says that no other coalition C⊊V can have a worth larger than the payoff *C* can receive by cooperating in *V*, and hence will not deviate from the grand coalition *V*. The core can be viewed as the stable payoff allocation. For a convex game, the core is always nonempty [17,25].

## 3. Problem Formulation

By regarding the set *V* of nodes as players, we consider a convex game with the characteristic function *g* being a supermodular function on $2^{V}$.

In particular, consider a weighted digraph on the set *V* of nodes. Such a graph can be characterized by the weight of the directed edges described using the weight function *w*:

$$w\left(B,C\right)=\sum_{i\in B,j\in C}a_{ij},$$

where $a_{i,j}$ is the weight of the edge from node *i* to node *j*. This covers the undirected graphs special cases when $a_{ij}=a_{ji}$ for all i,j∈V.

Consider the function *g* defined in the form of

(5) $$\begin{array}{c} g(B)=\gamma \cdot \beta \cdot w(B,B)-\gamma \cdot (1-\beta )\cdot w(V\setminus B,B), \end{array}$$

where β∈[0,1] and γ>0. The function *g* in (5) is supermodular [26]. When β=0, (5) reduces to the total weight of edges in *B* scaled by γ; when β=1, (5) reduces to the negative of the total weight of incoming edges from outside to *B* scaled by γ.

We want to identify strong communities based on the convex game using the following measure of community strength.

**Definition** **1.** *For C⊆V:|C|>1, define*

(6) σ(C):=minB⊊CB≠∅maxrC∈Core(C,g)mini∈Bri,

*which is referred to as the strength of community C.*

The inner maximization in (6) is the stable payoff guaranteed to any player in *B*, which we termed the community support to *B*. The outside minimization in (6) gives the strength of *C*, which is the minimum community support over B⊊C:B≠∅.

The following example illustrates the interpretation of the strength in (6) more concretely.

**Figure 1:**
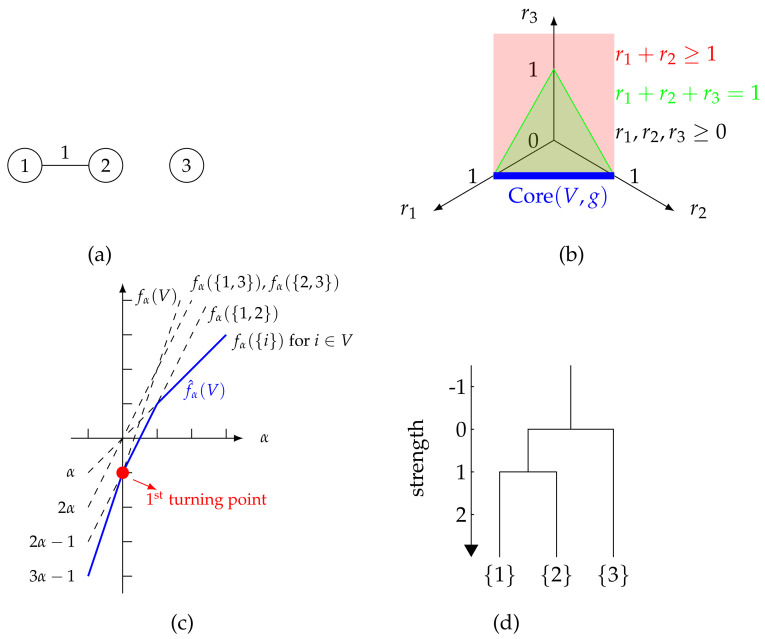
An illustrative example of an unweighted graph with $g\left(B\right):=\frac{1}{2}w\left(B,B\right)$ for B⊆V:B≠∅. (**a**) The unweighted graph; (**b**) Visualization of Core(V,g); (**c**) The curve ${\hat{f}}_{\alpha }\left(V\right)$; (**d**) The dendrogram.

**Example** **1.** *Consider the unweighted graph in Figure 1a with V={1,2,3} and characteristic function g in (5) with β=1 and γ=1, i.e., for B⊆V:B≠∅, $g\left(B\right)=\frac{1}{2}w\left(B,B\right)$, which calculates the total number of internal edges inside B.*

*We are going to show how to obtain the strength of {1,2}, which requires us to calculate the minimum community support over B⊊{1,2}:B≠∅ according to (6). By definition, the community support to B from {1,2} is the stable payoff that is guaranteed to each player in B. For a payoff allocation $r_{\left\{1,2\right\}}$ to be stable, it should be in Core({1,2},g), which is calculated to be*

(7) $$\begin{array}{c} Core\left(\{1,2\},g\right)=\{\left(r_{1},r_{2}\right)|r_{1}+r_{2}=1,r_{1}\geq 0,r_{2}\geq 0\}. \end{array}$$

*Then, we consider the guaranteed stable payoff to each player in B. For instance, when B={1}, the guaranteed stable payoff to players in B is 1, which is achieved with the payoff allocation $r_{\left\{1,2\right\}}=\left(1,0\right)$; when B={2}, the stable guaranteed payoff to players in B is 1, which is achieved with the payoff allocation $r_{\left\{1,2\right\}}=\left(0,1\right)$. Therefore, we know that the minimum community support to any non-empty proper subset of {1,2} is 1, i.e., the strength of {1,2} is 1.*

*Additionally, there is 1 unit of payoff that is transferable between players 1 and 2 based on the constraint for the core. Such a transferable payoff tends to improve the guaranteed payoff for players in non-empty subsets of C. As a result, {1,2} intuitively forms a meaningful community.*

*Similarly, we can show that the strength of V is 0. For a payoff allocation $r_{V}$ to be stable, it has to be in Core(V,g) which is calculated to be (see Appendix A.1)*

(8) $$\begin{array}{c} Core\left(V,g\right)=\{r_{V}:=\left(r_{1},r_{2},r_{3}\right)|r_{1}+r_{2}\geq 1,r_{1}\geq 0,r_{2}\geq 0,r_{3}=0\}. \end{array}$$

*Then, we consider the community support to B⊊V:B≠∅. For instance, when B={1}, the community support to B is 1, which is achieved with the payoff allocation $r_{V}=\left(1,0,0\right)$. By enumeration over B⊊V:B≠∅, we can obtain that when B={3}, the community support to B is 0, which is the minimum value of such community support. Hence, the strength of V is 0.*

There is another equivalent definition for σ(C) with (1) where we consider the average payoff allocated to a set B⊊C:B≠∅ instead of the inner minimization term $\min_{i\in B}r_{i}$ in (1), as stated in the following result.

**Proposition** **1.** *For C⊆V:|C|>1,*

(9) σ(C)=minB⊊C:B≠∅maxrC∈Core(C,g)r(B)|B|,

**Proof.**  See Appendix A.2. □

Our goal is to identify strong communities defined using σ as follows.

**Definition** **2.** *For any threshold α, define the collection of strong communities in V as*

(10) $$\begin{array}{c} C_{\alpha }\left(V\right):=\mathrm{maximal}\{C\subseteq V||C|>1,\sigma (C)>\alpha \}. \end{array}$$

The maximalF means inclusion-wise maximal subsets in F, i.e.,

(11) $$\begin{array}{c} \mathrm{maximal}F:=\{B\in F|∄C\in F,B⊊C\}. \end{array}$$

Similarly, minimalF means inclusion-wise minimal subsets in F, i.e.,

(12) $$\begin{array}{c} \mathrm{minimal}F:=\{B\in F|∄C\in F,B⊋C\}. \end{array}$$

**Example** **2.** *In Example 1, we already get σ(V)=0. Similarly, we can get σ({1,2})=1, σ({1,3})=0 and σ({2,3})=0.*

*According to (10), the strong communities in V given by our approach are*

(13) $$\begin{array}{c} C_{\alpha }\left(V\right)=\left\{\begin{array}{cc} \{V\}, & \alpha <0,\\ \{\{1,2\}\}, & \alpha \geq 0. \end{array}\right. \end{array}$$

## 4. Main Results

### 4.1. Characterization of Community Strength

The community strength defined in (9) takes a simpler form for the convex game as shown in Theorem 1.

**Theorem** **1.** *For any C⊆V:|C|>1,*

(14) σ(C)=minB⊊C:B≠∅g(C)−g(B)|C∖B|,

*Furthermore, the set of optimal solutions to (9) is given by*

(15) $$\begin{array}{c} \{C\setminus B|B\in S(C)\}, \end{array}$$

*where S(C) is the set of optimal solutions to the minimization in *(14)*.*

**Proof.**  See Appendix A.3. □

Equation (14) is the basic formula of community strength that we will utilize to derive the properties of the strong communities and investigate how to calculate the strong subsets.

The following example shows the equivalent value of the strength of *V* calculated by (9) and (14).

**Example** **3.** *Consider V as in Example 1, follow *(14)* to calculate the strength of V,*

(16)      σ(V)=minB⊊V:B≠∅g(V)−g(B)|C∖B|

(17) $$\begin{array}{cc} \; & =0 \end{array}$$

*with S(V)={{1,2}}. The value of σ(V) calculated here according to *(14)* is consistent with that calculated in Example 1.*

Define for α∈R and C⊆V:|C|>1 that

(18) f^α(C):=minB⊆C:B≠∅fα(B),where

(19) $$\begin{array}{cc} \;\;f_{\alpha }\left(B\right) & :=\alpha |B|-g(B),\;\mathrm{for}\;B\subseteq V. \end{array}$$

Denote the optimal solution set to (18) as $B_{\alpha }\left(C\right)$, and the collection of inclusion-wise minimal sets among $B_{\alpha }\left(C\right)$ as $B_{\alpha }^{*}\left(C\right)$, i.e.,

(20) $$\begin{array}{c} B_{\alpha }^{*}\left(C\right):=\mathrm{minimal}\;B_{\alpha }\left(C\right). \end{array}$$

$B_{\alpha }\left(C\right)$ is the set that we use to analyze the relation between σ(C) and the curve ${\hat{f}}_{\alpha }\left(C\right)$, and $B_{\alpha }^{*}\left(C\right)$ is the set we use for showing the computation of $C_{\alpha }\left(V\right)$ in the latter part.

The following example shows the curve of ${\hat{f}}_{\alpha }\left(V\right)$ for the set *V* in Example 1.

**Example** **4.** *Consider V as in Example 1, according to *(18)*,*

(21) $$\begin{array}{c} {\hat{f}}_{\alpha }\left(V\right)=\left\{\begin{array}{cc} 3\alpha -1, & \alpha <0,\\ 2\alpha -1, & 0\leq \alpha <1,\\ \alpha , & \alpha \geq 1, \end{array}\right. \end{array}$$

*and the inclusion-wise minimal solution set to *(18)* is given by*

(22) $$\begin{array}{c} B_{\alpha }^{*}\left(V\right)=\left\{\begin{array}{cc} \{V\}, & \alpha <0,\\ \{\{1,2\}\}, & 0\leq \alpha <1,\\ \{\{i\}|i\in V\}, & \alpha \geq 1, \end{array}\right. \end{array}$$

as illustrated in Figure 1c, where the result for ${\hat{f}}_{\alpha }\left(V\right)$ and $B_{\alpha }^{*}\left(V\right)$ can be obtained directly after we draw every curve of $f_{\alpha }\left(B\right)$ for B⊆V:B≠∅.

*For instance,*

- when α∈(0,1), ${\hat{f}}_{\alpha }\left(V\right)$ is given by $f_{\alpha }\left(\{1,2\}\right)$, hence $B_{\alpha }^{*}\left(V\right)=\{\{1,2\}\}$.
- when α=0, both {1,2} and V are solutions to *(18)* with respect to ${\hat{f}}_{\alpha }\left(V\right)$, while {1,2} is the inclusion-wise minimal solution, hence $B_{\alpha }^{*}\left(V\right)=\{1,2\}$.

From (19), it can be seen that $f_{\alpha }\left(B\right)$ is a linear function of α with slope |B|. Therefore, ${\hat{f}}_{\alpha }\left(C\right)$ in (18) is a piece-wise linear function since it is a minimization of linear functions. With C⊆V:|C|>1, the curve must have at least one turning point since the slope of $f_{\alpha }\left(\{i\}\right),i\in V$ is different from $f_{\alpha }\left(C\right)$. Figure 1c is the curve of ${\hat{f}}_{\alpha }\left(V\right)$ for *V* in Example 1.

The following result shows that σ(C) can be obtained from the curve. It will be used for deriving the representation and computation of the strong communities defined in Definition 2.

**Proposition** **2.** *For the curve ${\hat{f}}_{\alpha }\left(C\right)$ against α∈R:*

*(1)*  *σ(C) is the α-coordinate of the first turning point. More precisely,*
(23) minB⊊C:B≠∅fα(B)=fα(C)⇔σ(C)=α,
(24) minB⊊C:B≠∅fα(B)>fα(C)⇔σ(C)>α.
*(2)*  *The collection $B_{\alpha }\left(C\right)$ of optimal solution to *(18)* satisfies*
(25) $$\begin{array}{cc} B_{\alpha }\left(C\right) & ∌C,\;for\;\alpha >\sigma (C), \end{array}$$
(26) $$\begin{array}{cc} \;\;\;B_{\alpha }\left(C\right) & =S(C)\cup \{C\},\;for\;\alpha =\sigma (C), \end{array}$$
(27) $$\begin{array}{cc} B_{\alpha }\left(C\right) & =\{C\},\;for\;\alpha <\sigma (C). \end{array}$$

**Proof.**  See Appendix A.4. □

The following example can further illustrate the property of ${\hat{f}}_{\alpha }\left(V\right)$.

**Example** **5.** *In Example 4, the first turning point of the curve ${\hat{f}}_{\alpha }\left(V\right)$ is (0,−1), whose α-coordinate is exactly σ(V).*

### 4.2. Representation of Strong Communities

The strong communities defined in Definition 2 form a hierarchy and can be represented by a dendrogram.

**Theorem** **2.** *For any $C_{1}\in C_{\alpha_{1}}\left(V\right),C_{2}\in C_{\alpha_{2}}\left(V\right)$ where $\alpha_{1}\leq \alpha_{2}$, we have*

$$\begin{array}{c} C_{1}⊇C_{2},\;or\;C_{1}\cap C_{2}=\emptyset . \end{array}$$

*Furthermore,*

$$\begin{array}{c} if\;C_{1}⊋C_{2},\;then\;\alpha_{1}<\alpha_{2}. \end{array}$$

**Proof.**  See Appendix A.6. □

The following example shows that the strong communities in Figure 1a as in Example 1 with respect to two different α’s have a containment relationship.

**Example** **6.** *Let $\alpha_{1}:=-1$ and $\alpha_{2}:=1$. By the calculation results in Example 2, $C_{1}:=V\in C_{\alpha_{1}}\left(V\right)$ and $C_{2}:=\{1,2\}\in C_{\alpha_{2}}\left(V\right)$. Then $C_{1}⊋C_{2}$, which means the communities in $C_{\alpha_{1}}\left(V\right)$ are contained by those in $C_{\alpha_{2}}\left(V\right)$. This shows the hierarchical structure of the strong communities with respect to the specific $\alpha_{1}$ and $\alpha_{2}$.*

Theorem 2 follows from the following lemma.

**Lemma** **1.** *For all $C_{1},C_{2}\subseteq V:C_{1}\cap C_{2}\neq \emptyset$,*

(28) $$\begin{array}{c} \sigma \left(C_{1}\cup C_{2}\right)\geq \min\{\sigma \left(C_{1}\right),\sigma \left(C_{2}\right)\}. \end{array}$$

**Proof.**  See Appendix A.5. □

**Example** **7.** *As an example for showing Lemma 1, consider Figure 1a as in Example 1, let $C_{1}:=\{1,2\}$ and $C_{2}:=\{2,3\}$, then $C_{1}\cap C_{2}\neq \emptyset$. By the calculation results in Example 2,*

$$\begin{array}{c} \sigma \left(C_{1}\cup C_{2}\right)=0\geq \min\{\sigma \left(C_{1}\right),\sigma \left(C_{2}\right)\}, \end{array}$$

*i.e., *(28)* holds for $C_{1}$ and $C_{2}$.*

Lemma 1 establishes that the strength of the union of any two overlapping non-empty sets is lower bound by the smaller strength among the two sets, and this is the basis for Theorem 2.

The family $\underset{\alpha \in R}{⋃}C_{\alpha }\left(V\right)$ is said to be laminar and can be shown to contain at most |V|−1 elements. More precisely, we will show that the family of communities, together with their levels of strength, can be represented by the following dendrogram with σ, meaning the cophenetic similarity.

**Definition** **3.** *The dendrogram for the set of communities is defined as follows:*

*(1)*  *Every $C\in \underset{\alpha \in R}{⋃}C_{\alpha }\left(V\right)$ is an internal node annotated with the value σ(C);*
*(2)*  *Every singleton {i} for i∈V is a leaf node (annotated with the value +∞);*
*(3)*  *The parent of each node B⊊V:B≠∅ is defined as the minimum*
(29) $$\begin{array}{c} \mathrm{parent}\left(B\right):=\min\{C\in C_{\alpha }\left(V\right)|B⊊C,\alpha \in R\}. \end{array}$$

*As illustrated in Figure 2, the dendrogram forms a tree because each node (except the root node V) has a unique parent node.*

As a result of Theorem 2, the following corollary states that the parent of each strong community except *V* exists and is unique.

**Corollary** **1.** *For every B⊊V:B≠∅, the minimum element parent(B) exists.*

**Proof.**  See Appendix A.7. □

Using the following result, we can show that the set of children for each node $C\in \underset{\alpha \in R}{⋃}C_{\alpha }\left(V\right)$ is

$$C_{\sigma (C)}\left(C\right)\cup \{\{i\}\;|\;i\in V\setminus ⋃C_{\sigma (C)}\left(C\right)\},$$

which is also illustrated in Figure 2.

Analogous to Corollary 1, a community *B* has a parent *C* in the dendrogram if and only if *B* is in $C_{\sigma (C)}\left(C\right)$, and the strength of *B* is larger than that of *C*, as stated in the following corollary.

**Corollary** **2.** *For any nodes $C\in \underset{\alpha \in R}{⋃}C_{\alpha }\left(V\right)$ of the dendrogram,*

(30) $$\begin{array}{c} \mathrm{parent}\left(B\right)=C\Leftrightarrow B\in C_{\sigma (C)}\left(C\right), \end{array}$$

*which implies σ(B)>σ(C).*

**Proof.**  See Appendix A.8. □

**Example** **8.** *For Figure 1a as in Example 1, by the calculation results in Example 2, the dendrogram that corresponds to ${⋃}_{\alpha \in R}C_{\alpha }\left(V\right)$ is shown in Figure 1d.*

We defined the community strength in (6) by modeling the problem based on the convex game in game theory, gave its alternative forms in (9) and (14), and showed that the community strength and the solutions to the minimization of (14) are related to the first turning point of the curve defined by (18) against the parameter α. We also showed that the collection of strong communities defined in (10) form a hierarchy and can be represented by a dendrogram. These motivate the methods for computing strong communities, as described in the following section.

## 5. Computation of Strong Communities

In this section, we will show how to calculate the strong communities in $C_{\alpha }\left(V\right)$ at a threshold α, and how to calculate all the strong communities.

The following result shows that $C_{\alpha }\left(V\right)$ can be calculated with a recursive procedure.

**Theorem** **3.** *For α∈R, $C_{\alpha }\left(V\right)$ can be calculated with the following recurrence relation*

Cα(V)=(31)∅,if|V|≤1,(32)(Bα*(V)∖{{i}|i∈V})⋃Cα(U),otherwise,

*where*

(33) $$\begin{array}{cc} U & :=V\setminus ⋃B_{\alpha }^{*}\left(V\right), \end{array}$$

$B_{\alpha }^{*}\left(V\right)$ is defined in *(20)*, and *(31)* is the base case.

**Proof.**  See Appendix A.9. □

Theorem 2 shows that $C_{\alpha }\left(V\right)$ can be computed in a divisive way. In the first recursive step, *V* is the ground set, if |V|≤1, we directly calculate $C_{\alpha }\left(V\right)=\emptyset$ by the base case (31) and stop the recursion; otherwise we calculate $B_{\alpha }^{*}\left(V\right)$, then $B_{\alpha }^{*}\left(V\right)\setminus \{\{i\}|i\in V\}$ is the set of newly found strong subsets, and we enter the next recursive step. The new recursive step is similar to the first recursive step, but we use *U* given in (33) as the ground set.

The following example shows how to run the recursive procedure in Theorem 3 for computing $C_{\alpha }\left(V\right)$.

**Example** **9.** *Consider Figure 1a as in Example 1 and we calculate $C_{\alpha }\left(V\right)$ at $\alpha =\frac{1}{2}$ by following Theorem 3.*

*(1)*  *The first recursive step:*
  - *|V|>1, which corresponds to the case in *(32)*.*
  - Then we need to compute $B_{\alpha }^{*}\left(V\right)$. By the calculation in Example 4, we know $B_{\alpha }^{*}\left(V\right)=\{\{1,2\}\}$.
  - By *(32)*, the elements in $B_{\alpha }^{*}\left(V\right)\setminus \{\{i\}|i\in V\}=\{\{1,2\}\}$ are in $C_{\alpha }\left(V\right)$, and computing $C_{\alpha }\left(U\right)$ will provide us the remaining strong subsets in $C_{\alpha }\left(V\right)$, where U is given by *(33)*. Here, U={3}.
*(2)*  *The second recursive step:*
  - *Regard U as the ground set and compute $C_{\alpha }\left(U\right)$ according to *(31)* and *(32)*.*
  - *Since |U|=1, the base case *(31)* applies, which means $C_{\alpha }\left(U\right)=\emptyset$, and the recursive procedure ends.*

*According to the recursive steps, $C_{\alpha }\left(V\right)=\{\{1,2\}\}$.*

*Notice that in this example, there are two recursive steps in total. For some other examples where the U obtained in the first recursive step has a cardinality larger than 1, i.e., the case *(32)* applies, then the following recursive step will be similar to the first recursive step except that U instead of V is regarded as the ground set.*

Additionally, we use the $B_{\alpha }^{*}\left(V\right)$ from the calculation in Example 4, which employs a brute force method enumerating all B⊆V:B≠∅. We will discuss how to compute $B_{\alpha }^{*}\left(V\right)$ in polynomial time later.

The following example illustrates why there are strong communities not in $B_{\alpha }^{*}\left(V\right)$ and why the recursive procedure in Theorem 3 can identify those strong communities.

**Figure 3:**
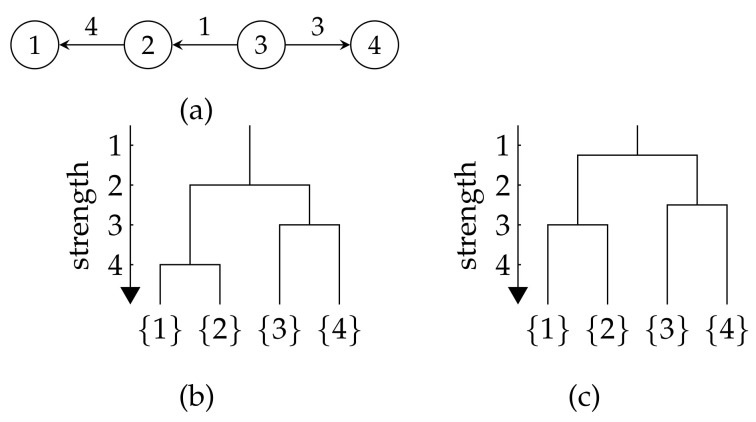
A simple digraph and the dendrogram when *g* is defined by (46) with different β. (**a**) The digraph; (**b**) The dendrogram when β=1; (**c**) The dendrogram when $\beta =\frac{1}{2}$.

**Figure 2:**
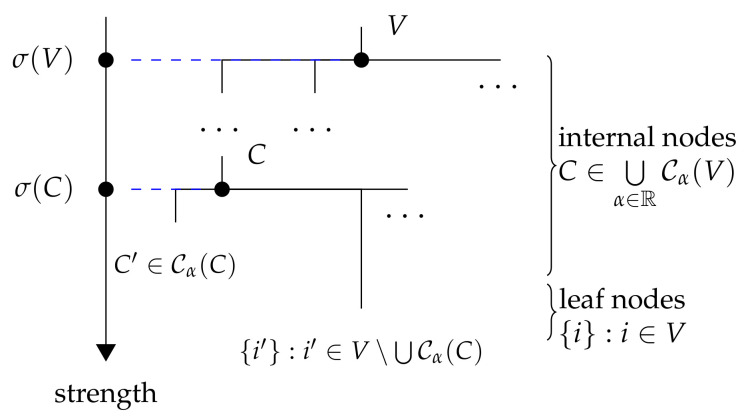
Dendrogram of the communities.

**Example** **10.** *Consider Figure 3a on V={1,2,3,4} with function g defined by *(5)* with γ=1 and $\beta =\frac{1}{2}$. Then g(C) is the total weight of internal edges in C.*

*In the graph, $C_{1}:=\{1,2\}$ and $C_{2}:=\{3,4\}$ have relatively large total weights of internal edges compared with other subsets of V hence they are meaningful communities that are expected to be identified.*

Let α=2. $B_{\alpha }^{*}\left(V\right)$ in *(20)* contains the minimal non-empty subsets of V that leads to the minimum value of $f_{\alpha }\left(\cdot \right)$ in *(19)*.

$C_{1}$ is identified by $B_{\alpha }^{*}\left(V\right)$ because $f_{\alpha }\left(C_{1}\right)$ achieves the minimum value among all the non-empty subsets of V. However, the other meaningful community $C_{2}$ is not in $B_{\alpha }^{*}\left(V\right)$ because $C_{2}$ will never be a subsets of V that leads to the minimum value of $f_{\alpha }\left(\cdot \right)$, as $f_{\alpha }\left(C_{1}\right)<f_{\alpha }\left(C_{2}\right)$ always holds. In other words, $C_{1}$ dominates $C_{2}$.

To identify $C_{2}$, we remove the nodes that appeared in the communities in *(32)* from V, as described in *(33)*, and then start a new recursive step to identify strong communities within the remaining nodes. Since the community $C_{1}$ that dominated $C_{2}$ in V was removed, $C_{2}$ can now be identified with $B_{\alpha }^{*}\left(\cdot \right)$. In this way, the recursive procedure in Theorem 3 works to identify all the strong communities in $C_{\alpha }\left(V\right)$.

For the recurrence relation in Theorem 3 to be applicable, the recursive procedure in Theorem 3 finishes with finite recursive steps. The following results imply that *U* in (31) always has a smaller size than *V*.

**Proposition** **3.** *For α∈R and the set V with |V|≥2,*

(34) $$\begin{array}{c} |B_{\alpha }^{*}\left(V\right)|\geq 1. \end{array}$$

**Proof.**  See Appendix A.10. □

As a result of Proposition 3, the number of recursive steps in Theorem 3 needed is bounded by |V|.

**Proposition** **4.** *For a non-empty set V, it takes at most |V| recursive steps to calculate $C_{\alpha }\left(V\right)$ by Theorem 3.*

**Proof.**  See Appendix A.11. □

The following property is the basis of Theorem 3, which ensures that the recursive procedure in Theorem 3 does not leave out any strong communities in $C_{\alpha }\left(V\right)$ for a chosen α.

**Proposition** **5.** For any $B\in B_{\alpha }^{*}\left(V\right):\left|C\right|>1$, ∀C⊆V,

(35) $$\begin{array}{cc} B\cap C\neq \emptyset \;and\;C⊈B & \Rightarrow \sigma (C)\leq \alpha , \end{array}$$

*or the contrapositive*

(36) $$\begin{array}{cc} \sigma (C)>\alpha & \Rightarrow B\cap C=\emptyset \;or\;C\subseteq B. \end{array}$$

**Proof.**  See Appendix A.12. □

Equation (36) implies that any other strong subset with strength larger than α is either disjoint with the elements in $B_{\alpha }^{*}\left(V\right)$, or is a subset of an element in $B_{\alpha }^{*}\left(V\right)$. This ensures that when we continue the computation with *U* in (33) as the ground set after we obtained the strong subset with strength larger than α in $B_{\alpha }^{*}\left(V\right)\setminus \{\{i\}|i\in V\}$, the remaining strong subsets will be captured by $C_{\alpha }\left(U\right)$.

To obtain $C_{\alpha }\left(V\right)$, calculating $B_{\alpha }^{*}\left(V\right)$ is a basic step that requires optimization of (18), which can be done based on the method in [26] as described in the following.

### 5.1. Divide-and-Conquer

We rewrite the minimization of ${\hat{f}}_{\alpha }\left(V\right)$ in (18) in a similar way as that in [26]:

(37) $$\begin{array}{cc} \;\;\;\;{\hat{f}}_{\alpha }\left(V\right) & =\min_{t\in V}{\hat{f}}_{\alpha }^{\left(t\right)}\left(V\right),\;\mathrm{where} \end{array}$$

(38) f^α(t)(V):=minB⊆V:t∈Bfα(B),

which is a two-step optimization problem, and denote $B_{\alpha }^{\left(t\right)}\left(V\right)$ as the minimal optimal solution set to (38).

Since ${\hat{f}}_{\alpha }$ defined in (18) is a submodular function, $B_{\alpha }^{\left(t\right)}\left(V\right)$ can be solved with submodular function minimization (SFM) algorithms, and it has a unique element since the feasible domain {B⊆V|j∈B} is a lattice ([27] Proposition 10.1).

Let $T_{\alpha }^{*}\left(V\right)$ be the set of optimal solutions *t* to (37), we have the following result that indicates how $B_{\alpha }^{*}\left(V\right)$ can be calculated.

**Proposition** **6**  ([26] Proposition 2). For α∈R, $B_{\alpha }^{*}\left(V\right)$ in *(20)* can be obtained from $B_{\alpha }^{\left(t\right)}\left(V\right),t\in V$ and $T_{\alpha }^{*}\left(V\right)$ by

(39) $$\begin{array}{c} B_{\alpha }^{*}\left(V\right)=\mathrm{minimal}\;\underset{t\in T_{\alpha }^{*}\left(V\right)}{⋃}B_{\alpha }^{\left(t\right)}\left(V\right). \end{array}$$

**Proof.**  See Appendix A.13. □

According to Theorem 3, computing $C_{\alpha }\left(V\right)$, all the strong subset with a strength larger than α, can be done with the following steps:

(1) Calculate $B_{\alpha }^{\left(t\right)}\left(V\right)$ for t∈V by optimizing (38) with SFM algorithms;
(2) Calculate $T_{\alpha }^{*}\left(V\right)$ by optimizing (38);
(3) Calculate $B_{\alpha }^{*}\left(V\right)$ according to (39);
(4) Calculate *U* by (33);
(5) M is the newly found strong subsets that have a strength larger than α in this recursive step. If |U|≤1, then stop; otherwise, regard *U* as *V* and go to (1) to start a new recursive step.

The union of the set of strong subsets calculated in all the recursive steps is $C_{\alpha }\left(V\right)$.

To calculate all the strong subsets, i.e., ${⋃}_{\alpha \in R}C_{\alpha }\left(V\right)$, for each t∈V, define

(40) $$\begin{array}{c} g^{\left(t\right)}\left(A\right):=g\left(A\cup \{t\}\right)-g\left(\{t\}\right)\;\mathrm{for}\;A\subseteq \left(V\setminus \{i\}\right), \end{array}$$

then

(41) $$\begin{array}{c} B^{\prime }\subseteq \left(V\setminus \{i\}\right)\mapsto \alpha \left|B^{\prime }\right|-g^{\left(t\right)}\left(B^{\prime }\right) \end{array}$$

is a normalized submodular function.

We need to obtain the minimal optimal solution to (41) for all α∈R. Luckily, with SFM algorithms such as Wolfe’s minimum norm point algorithm [28], for (41), we can obtain for some $N_{t}^{\prime }\in N$ the sequence of $\alpha_{1}^{\left(t\right)},\alpha_{2}^{\left(t\right)},\cdots ,\alpha_{N_{t}^{\prime }}^{\left(t\right)}$ and the corresponding sequence of sets $A_{1}^{\left(t\right)},A_{2}^{\left(t\right)},\cdots ,A_{N_{t}^{\prime }}^{\left(t\right)}$ that satisfies ([27] Proposition 8.6)

(42) $$\begin{array}{c} -\infty =\alpha_{0}^{\left(t\right)}<\alpha_{1}<\alpha_{2}^{\left(t\right)}<\cdots <\alpha_{N_{t}^{\prime }}^{\left(t\right)}<\alpha_{N_{t}^{\prime }+1}^{\left(t\right)}\infty , \end{array}$$

(43) $$\begin{array}{c} A_{0}^{\left(t\right)}:=V⊋A_{1}^{\left(t\right)}⊋A_{2}^{\left(t\right)}⊋\cdots ⊋A_{N_{t}^{\prime }}^{\left(t\right)}:=\emptyset , \end{array}$$

and for any $\alpha \in \left[\alpha_{i}^{\left(t\right)},\alpha_{i+1}^{\left(t\right)}\right),i\in \{0,1,\cdots ,N_{t}^{\prime }\},$

(44) $$\begin{array}{c} A_{i}^{\left(t\right)}\;\mathrm{is}\;\mathrm{the}\;\mathrm{minimal}\;\mathrm{minimizer}\;\mathrm{to}\;\left(41\right). \end{array}$$

Equation (44) means with the sequences (42) and (43), we can obtain the minimum solution to (41) for all α∈R.

For any α∈R, if $A^{\left(t\right)}$ is the unique minimal solution to (41), then $B^{\left(t\right)}=A^{\left(t\right)}\cup \{i\}$ will be the unique minimal solution to (37), or in another word,

(45) $$\begin{array}{c} B_{\alpha }^{\left(t\right)}\left(V\right)=\{B^{\left(t\right)}\}, \end{array}$$

since $g\left(B^{\prime }\right)-g^{\left(t\right)}\left(B^{\prime }\right)=g\left(\{t\}\right)$ is a constant for all C⊆V∖{t}. This means the minimal solution set $B_{\alpha }^{\left(t\right)}\left(V\right)$ to (37) for all α∈R can be obtained from the solutions to (41) with sequences (42) and (43).

Therefore, with sequences (42) and (44) for all t∈V, $T_{\alpha }^{*}\left(V\right)$ can be obtained for all α∈R. Then calculating $C_{\alpha }\left(V\right)$ for all $\alpha \in {⋃}_{t\in V}\{a_{0}^{\left(t\right)},\alpha_{1},\cdots ,\alpha_{N_{t}^{\prime }+1}^{\left(t\right)}\}$ based on Theorem 3, Proposition 6 and $T_{\alpha }^{*}\left(V\right)$ is sufficient for us to obtain ${⋃}_{\alpha \in R}C_{\alpha }\left(V\right)$.

With MNB(n) denoting the complexity of the minimum norm base algorithm for SFM on the ground set of size *n*, we have the following result.

**Proposition** **7.** *${⋃}_{\alpha \in R}C_{\alpha }\left(V\right)$ can be computed in ${O(|V|}^{3}\mathrm{MNB}(|V|))$ time.*

**Proof.**  See Appendix A.14. □

### 5.2. Using Max-Flow Min-Cut Algorithm

For the step of optimizing (38) in computing $C_{\alpha }\left(V\right)$, SFM algorithms are used. However, SFM algorithms are generally computationally expensive. There are works on improving the efficiency of SFM problems by max-flow min-cut algorithms [29,30]. We discuss a category of choices for *g* when the max-flow min-cut algorithm can be utilized for computing $C_{\alpha }\left(V\right)$.

Consider the function *g* defined in the form of ([26] Difinition 2)

(46) $$\begin{array}{c} g(B)=\beta \cdot w(B,B)-(1-\beta )\cdot w(V\setminus B,B), \end{array}$$

which is a special case of (5) with γ=1.

Following the method in [26], we can construct an augmented digraph and run a max-flow min-cut algorithm [31,32,33,34] to obtain the solution to (38). With α in (19) and β in (46), the (α,β,t)-augmented digraph [26] is a digraph on {V}∪{s} where s∉V is an additional node, with the edge weight $w_{\alpha ,\beta ,t}:\left(V\cup \{i\}\right)\to R_{\geq 0}$ defined as

(47) $$\begin{array}{c} w_{\alpha \beta t}\left(i,j\right):=\left\{\begin{array}{cc} w(i,j), & i\in V,j\in V\setminus \{t\}\\ w\left(i,j\right)+\beta d_{i}, & i\in V,j=t\\ \alpha , & i=s,j\in V\\ 0, & \mathrm{otherwise}. \end{array}\right. \end{array}$$

**Proposition** **8**  ([26] Theorem 3). *The $B_{\alpha }^{\left(t\right)}\left(V\right)$ contains a unique minimum set C such that $\left(\{s\}\cup V\setminus C,C\right)$ is a minimum s-t cut of the (α,β,t)-augmented digraph.*

Proposition 8 implies that $B_{\alpha }^{\left(t\right)}\left(V\right)$ can be solved by max-flow min-cut algorithm. Moreover, with the parametric max-flow min-cut algorithms [34], we can obtain $B_{\alpha }^{\left(t\right)}\left(V\right)$ for all α≥0. Hence, when *g* has the form of (46), computing $C_{\alpha }\left(V\right)$ for a certain α or all α follows the same procedure in Section 5.1, except that we can use max-flow min-cut algorithms to calculate $B_{\alpha }^{\left(t\right)}\left(V\right)$ instead of SFM algorithms.

## 6. Discussions

To illustrate the dendrogram of strong communities found by our approach, the digraph in Figure 3a is used as an example, with the function *g* given in (46) for different choices of β for experiments.

The result for the cases β=1 and β=0.5 are shown in Figure 3b and Figure 3c, respectively. The example of the calculation procedures based on Theorem 3 for the case β=1 is in Appendix A.15.

We can obtain the collection of strong communities $C_{\alpha }\left(V\right)$ in (10) for α from the dendrogram. For instance, the strong communities for $\alpha =\frac{5}{2}$ is,

$$C_{\frac{5}{2}}\left(V\right)=\left\{\{1,2\},\{3,4\}\right\}.$$

The parameter β in (46) is a balancing factor between the total weight of internal edges and the negative total weight of incoming edges, and when β=1, it can be used for the problem of finding the minimal densest subgraphs.

In [35], another kind of augmented graph is constructed, and an algorithm is given for quickly increasing α value based on the current community that has already been found and then conducting max-flow min-cut algorithms. We want to point out that, although the method there is similar to solving $B_{\alpha }\left(V\right)$ in our work, the algorithm there for calculating the next critical α, as the author also said, needs more calculation steps if we want to obtain more solutions for intermediate $\alpha^{\prime }s$. In other words, not all critical $\alpha^{\prime }s$ are found, while our approach calculates all the critical $\alpha^{\prime }s$ and the solutions directly. Additionally, our approach goes beyond just finding $B_{\alpha }\left(V\right)$, and we considered digraphs, which can be generalized to undirected graphs directly.

The work in [26] extends the notion of web communities [36] to digraphs and calculates the communities in polynomial time, which is closely related to our approach. In fact, $B_{\alpha }^{*}\left(V\right)\setminus \{\{i\}|i\in V\}$ is the set of web communities, which is included in the strong communities defined in (10) in this work. For a set C⊊V:C≠∅, subsets of V∖C can prevent *C* from being a web community in [26], even if *C* has a strength larger than α. Nevertheless, such a phenomenon does not exist for strong communities detected by our approach. Whether *C* is a strong community in (10) or not is independent of subsets of V∖C according to (10). In Example 10, $C_{1}$ is a web community, and $C_{2}$ is not, since $C_{2}$ is dominated by $C_{1}$. However, $C_{2}$ is a meaningful community that is expected to be identified. The web community method fails to identify $C_{2}$, while our approach can identify it as a strong community, as we have calculated in the example.

**Figure 4:**
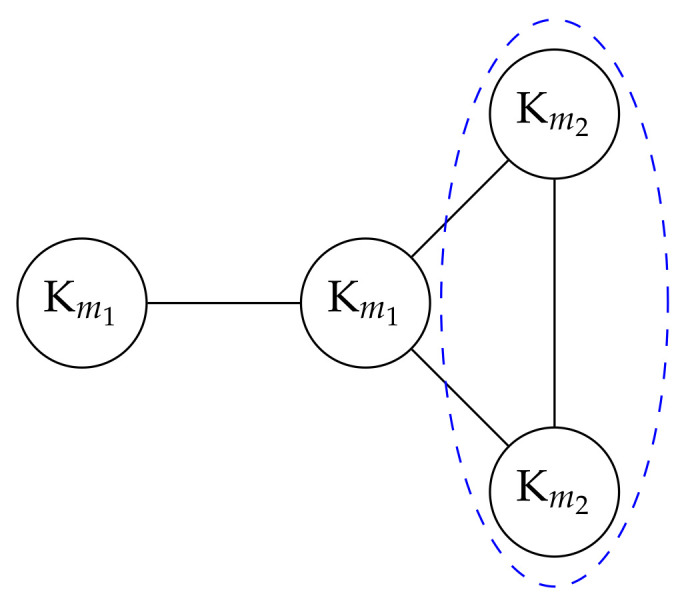
An unweighted graph with $m_{1}=20$, $m_{2}=5$ where $K_{m}$ denotes the complete graph with *m* nodes [38]. As for the two smaller complete graphs denoted by $K_{m_{2}}$, Modularity [37] will merge the two into a single community as indicated by the blue dashed ellipse due to resolution limit [38], while our approach can identify each of them as strong communities.

Our approach also addresses some known issues associated with existing community detection methods. For instance, Modularity [37], a common community detection method, is NP-hard and suffers from the limitation of resolution limit [38]. There are works such as [39,40] aiming at resolving the resolution limit issue, yet both rely on heuristics to obtain solutions. It is worth mentioning that Modularity is a measure applied over partitions, while our strength measure is on individual communities. In Figure 4, there are four complete graphs of two sizes, $m_{1}=20$ and $m_{2}=5$. Despite the two complete graphs of size $m_{2}$ being smaller than the other two, they should be identified as communities since they are the maximal complete graphs. However, Modularity fails to recognize the two smaller complete graphs, and instead, it merges them into a single community [38]. In contrast, our approach successfully identifies the two smaller complete graphs of size $m_{2}$ as strong communities.

The strong communities are derived from game theory, where the strength can be interpreted as the support inside the community that can be shared with a part of individuals in need in the same community. Application in real-world problems is promising, such as finding small groups of advertisers and keywords in sponsored auctions, where the community strength means the average money inside the groups [35,41].

## 7. Conclusions

We introduced a novel concept of strength for community detection using a convex game model in cooperative game theory. It can be applied to networks with a supermodular characteristic function. Theorem 1 establishes the dual objective function, based on which we conducted a comprehensive analysis of strong community properties. The laminar structure demonstrated in Theorem 2 reveals that strong communities form a hierarchy and can be represented by a dendrogram.

To compute strong subsets, Theorem 3 introduces a recurrence relation, enabling polynomial time calculations through submodular function minimization. For specific characteristic function choices in the convex game on the graphical model, an augmented digraph can be constructed to apply the max-flow min-cut algorithm to improve computation efficiency.

Unlike many existing community detection methods, which often rely on approximation, are non-deterministic, and lack guarantees on complexity, our approach for community detection is deterministic, computable in polynomial time, and supported by a rigorous theoretical analysis of its properties. Since our approach captures high-order statistics through the supermodular characteristic function, the primary limitation of our approach lies in its computational complexity. This complexity presents a challenge when applying the method to large-scale real-world datasets. Nevertheless, our work proposes an analytical framework for community detection that yields unique solutions and provides theoretical foundations for future research aimed at improving the complexity and empirical applications.

## Data Availability

Data are contained within the article.

## Appendix A

### Appendix A.1

We show the computation and visualization of Core(V,g) in Example 1.

According to (4),

(A1)Core(V,g)={rV:=(r1,r2,r3)|r1+r2+r3=g(V)=1,(A2)               r1+r2≥g({1,2})=1,(A3)               r1+r3≥g({1,3})=0,(A4)               r2+r3≥g({2,3})=0,(A5)          r1,r2,r3≥0}(A6)     ={(r1,r2,r3)|r1+r2≥1,r1≥0,r2≥0,r3=0},

where $r_{1},r_{2},r_{3}$ are the payoff allocated to players 1,2 and 3, respectively.

Equation (A1) ensures that the payoff vector $r_{V}$ exactly splits the total worth of *V*, (A2) ensures that the total payoff for {1,2} is no less than the worth of {1,2} so that players in {1,2} will not deviate from *V*. The interpretation for (A3)–(A5) is similar to (A2). The intersection defined by (A1) through (A5) is the Core(V,g) which is visualized in Figure 1b, where

- (A1) is the region given by the plane containing (1,0,0),(0,1,0) and (0,0,1), as indicated by green color;
- (A2) is the region that includes the plain and on the opposite side of the origin relative to the plane, which contains (1,0,0) and (0,1,0) and parallel to $r_{3}$ axis, as indicated by red color;
- (A5) restricts each element of $r_{V}$ to be non-negative, which includes the region represented by (A3) and (A4); and
- the line segment in blue between (1,0,0) and (0,1,0) is Core(V,g).

Equation (A6) is the simplified algebraic form for Core(V,g).

### Appendix A.2

**Proof** **of** **Proposition** **1.** Given C⊆V:|C|>1, for any B⊊C:B≠∅ and payoff vector $r_{C}$,

(A7) $$\begin{array}{c} \min_{i\in B}r_{i}\leq \frac{r(B)}{\left|B\right|}. \end{array}$$

Then, we show that the equality in (A7) holds. Suppose node j∈B is a solution to the left-hand side of (A7), i.e.,

(A8) $$\begin{array}{c} \min_{i\in B}r_{i}=r_{j}. \end{array}$$

Let B={j}, then the right-hand side of (A7) is

(A9) $$\begin{array}{c} \frac{r(B)}{\left|B\right|}=r_{j}. \end{array}$$

Equations (A8) and (A9) imply that the equality in (A7) holds. Since the equality in (A7) holds for B⊊C:B≠∅ and payoff vector $r_{C}$, then

(A10) minB⊊CB≠∅maxrC∈Core(C,g)mini∈Bri=minB⊊CB≠∅maxrC∈Core(C,g)r(B)|B|,

which establishes (9) according to (6). □

### Appendix A.3

**Proof** **of** **Theorem** **1.** For ∀B⊊C,B≠∅, by the relationship between r(·) and g(·) when $r_{C}\in \mathrm{Core}\left(C,g\right)$, we have

r(B)=r(C)−r(C∖B)≤g(C)−g(C∖B),

where the equality is achieved when r(C∖B)=g(C∖B). Hence,

(A11) $$\begin{array}{cc} \max_{r_{C}\in \mathrm{Core}\left(C,g\right)}\frac{r(B)}{\left|B\right|} & \leq \frac{g(C)-g(C\setminus B)}{\left|B\right|} \end{array}$$

Next, we will show that $\exists r_{C}\in \mathrm{Core}\left(C,g\right)$ s.t. r(C∖B)=g(C∖B) so that the equality is achieved in (A11). To ease the notation, denote

(A12) $$\begin{array}{c} T:=C\setminus B. \end{array}$$

With *T*, (A11) becomes

(A13) $$\begin{array}{cc} \max_{r_{C}\in \mathrm{Core}\left(C,g\right)}\frac{r(B)}{\left|B\right|} & \leq \frac{g(C)-g(T)}{\left|C\setminus T\right|} \end{array}$$

For the convex subgame (T,g), the core of (T,g) is non-empty since the game is convex [17,25]. Suppose $r_{T}\in \mathrm{Core}\left(T,g\right)$, which means

(A14) $$\begin{array}{cc} r(T) & =g(T),\mathrm{and} \end{array}$$

(A15) $$\begin{array}{cc} \;\;r(T^{\prime }) & \geq g\left(T^{\prime }\right),\forall T^{\prime }\subseteq T. \end{array}$$

Then we show the steps to construct a vector $r_{C}$ from $r_{T}$ with $r_{C}\in \mathrm{Core}\left(C,g\right)$. Step 1: Select an *i*, with

(A16) $$\begin{array}{c} i\in \underset{j:j\in C\setminus T}{\arg\max\;}g\left(T\cup \left\{j\right\}\right)-g\left(\left\{j\right\}\right). \end{array}$$

Step 2: Assign

(A17) $$\begin{array}{c} r_{i}=g\left(T\cup \left\{i\right\}\right)-g\left(\left\{i\right\}\right), \end{array}$$

and construct $r_{T\cup \{i\}}$ by values from $r_{T}$ and $r_{i}$. For $\forall T^{\prime }\subseteq T$, by supermodularity of *g*,

(A18) $$\begin{array}{c} g\left(T^{\prime }\cup \left\{i\right\}\right)-g\left(T^{\prime }\right)\leq g\left(T\cup \left\{i\right\}\right)-g\left(T\right). \end{array}$$

Then,

(A19) r(T′∪{i})=r({i})+r(T′)(A20)     =g(T∪{i})−g({i})+r(T′)(A21)     ≥g(T∪{i})−g({i})+g(T′)(A22)≥g(T′∪{i}),

where (A19) is by (1), (A20) is by (A17), (A21) is by (A15), and (A22) is by (A18). Equations (A22), (A14) and (A15) indicate that

(A23) $$\begin{array}{c} r_{T\cup \{i\}}\in \mathrm{Core}\left(T\cup \left\{i\right\},g\right). \end{array}$$

Update *T* by T∪{i}, and continue the Step 1 and Step 2 above, and finally we will have a constructed

(A24) $$\begin{array}{c} r_{C}\in \mathrm{Core}\left(C,g\right), \end{array}$$

with r(T)=g(T) preserved. This means, for ∀B⊊C,B≠∅, the formula (A11), or its equivalent formula (A13), can achieve equality with the constructed $r_{C}$ obtained in (A24). Then (14) is implied considering (9). Therefore, the minimization over the non-empty proper subset of *C* for the right-hand side of (A13) will lead to a value equal to the minimization over the non-empty proper subset of the left-hand side of (A13), which is the community strength for *C* defined in (9). And based on the above proof, we know that if a non-empty set $B^{*}$ is a solution for the minimization in (9), the set $T^{*}:=C\setminus B^{*}$ will be a solution for the minimization in (14). Hence, Theorem 1 is established. □

### Appendix A.4

**Proof** **of** **Proposition** **2.** We first show the second property in Proposition 2, which will imply the first property. Denote the sign of a number *x* by

(A25) $$\begin{array}{c} sgn\left(x\right):=\left\{\begin{array}{c} 1,\;x>0\\ -1,\;x<0\\ 0,\;x=0. \end{array}\right. \end{array}$$

We have

$$\begin{array}{cc} sgn(\sigma (C) & -\alpha ) \end{array}$$

(A26a) =sgn(minB⊊C:B=∅g(C)−g(B)|C∖B|−α)

(A26b)  =sgn(minB⊊C:B=∅g(C)−g(B)−α|C∖B|)

(A26c) =sgn(minB⊊C:B=∅fα(B)−fα(C)),

where

- (A26a) is by (14);
- (A26b) is because |C∖B|>0 and B≠C and sgn(x)=sgn(ax) if a>0;
- (A26c) is by the definition of $f_{\alpha }$ in (19).

Note also that the sets of optimal solutions to the minimization/maximization in each step are the same. Hence,

(A27) $$\begin{array}{cc} \sigma (C)<\alpha & \Leftrightarrow sgn(\sigma (C)-\alpha )<0 \end{array}$$

(A28) ⇔sgn(minB⊊C:B=∅fα(B)−fα(C)}<0

(A29)    ⇔minB⊊C:B=∅fα(B)<fα(C)

(A30) $$\begin{array}{cc} & \;\Leftrightarrow C\notin B_{\alpha }\left(C\right), \end{array}$$

where

- (A27) is by (A25);
- (A28) is by (A26c);
- (A29) is by (A25);
- (A30) is by definition of $B_{\alpha }\left(C\right)$.

Equation (A30) implies (25). Similarly, with the inequalities “<” replaced by “>”, and simply by definition of $B_{\alpha }\left(C\right)$, we have

σ(C)>α⇔minB⊊C:B=∅fα(B)>fα(C)⇔{C}=Bα(C),

which implies (24) and (27). With the inequalities replaced by equalities, we have

σ(C)=α⇔minB⊊C:B=∅fα(B)=fα(C),

which implies (23) and (26). This completes the proof. □

### Appendix A.5

**Proof** **of** **Lemmma** **1.** Assume

(A31) $$\begin{array}{c} \sigma \left(C_{1}\right)\geq \sigma \left(C_{2}\right)=\alpha \end{array}$$

It suffices to show that $\sigma (C_{1}\cup C_{2})\geq \alpha$, or equivalently, by (23) and (24),

(A32) $$\begin{array}{c} f_{\alpha }\left(B\right)\geq f_{\alpha }\left(C_{1}\cup C_{2}\right),\forall B\subseteq C_{1}\cup C_{2}:B\neq \emptyset . \end{array}$$

Consider the case $B\subseteq C_{1}$ first,

$$\begin{array}{cc} f_{\alpha }\left(B\right) & \overset{\left(a\right)}{\geq }f_{\alpha }\left(C_{1}\right)\\ \; & \overset{\left(b\right)}{\geq }f_{\alpha }\left(C_{1}\cup C_{2}\right)\underset{\overset{\left(c\right)}{\geq \;0}}{\underset{︸}{-f_{\alpha }\left(C_{1}\right)+f_{\alpha }\left(C_{1}\cap C_{2}\right)}} \end{array}$$

which implies (A32) as desired, where

- (a) and (c) are by (23) and (24), since both $\sigma (C_{1})$ and $\sigma (C_{2})$ are at least α by (A31);
- (b) is because $f_{\alpha }$ is submodular due to the supermodularity of *g* and modularity of the cardinality function.

Then consider the remaining case $B⊈C_{1}$, i.e., $B\subseteq C_{1}\cup C_{2}$ and $B\cap C_{2}\neq \emptyset$,

$$\begin{array}{cc} f_{\alpha }\left(B\right) & \overset{\left(d\right)}{\geq }f_{\alpha }\left(B\cup C_{2}\right)\underset{\overset{\left(e\right)}{\geq \;0}}{\underset{︸}{-f_{\alpha }\left(C_{2}\right)+f_{\alpha }\left(B\cap C_{2}\right)}}\\ \; & \overset{\left(f\right)}{\geq }f_{\alpha }(\underset{\overset{g}{=}C_{1}\cup C_{2}}{\underset{︸}{\left(B\cup C_{2}\right)\cup C_{1})}}\underset{\overset{\left(h\right)}{\geq \;0}}{\underset{︸}{-f_{\alpha }\left(C_{1}\right)+f_{\alpha }(\left(B\cup C_{2}\right)\cap C_{1}}}, \end{array}$$

which implies (A32) as desired, where

- (d) and (f) are by the submodularity of $f_{\alpha }$ explained above;
- (h) is by (23) and (24) since $\left(B\cup C_{2}\right)\cap C_{1}\subseteq C_{1}$, $\left(B\cup C_{2}\right)\cap C_{1}⊇C_{1}\cap C_{2}$ and $C_{1}\cap C_{2}\neq \emptyset$ by the assumption stated in the lemma;
- (g) is because $B\subseteq C_{1}\cup C_{2}$.

□

### Appendix A.6

**Proof** **of** **Theorem** **2.** Suppose on the contrary that $C_{1}⊉C_{2}$ and $C_{1}\cap C_{2}\neq \emptyset$. By Lemma 1,

$$\begin{array}{c} \sigma \left(C_{1}\cup C_{2}\right)\geq \sigma \left(C_{1}\right)>\alpha_{1}. \end{array}$$

This contradicts $C_{1}\in C_{\alpha_{1}}\left(V\right)$, in particular, the inclusion-wise maximality of $C_{1}$, since $C_{1}\cup C_{2}⊋C_{1}$, where the strict inclusion is because $C_{1}⊉C_{2}$. □

### Appendix A.7

**Proof** **of** **Corollary** **1.** Consider B⊊V:B≠∅. An inclusion-wise minimal element in the set in its parent p(B) exists since V is in the set. Suppose on the contrary that there can be multiple minimal elements, say $C_{1}\in C_{\alpha }\left(V\right)$ and $C_{2}\in C_{\alpha }\left(V\right)$ with $\alpha_{1}\leq \alpha_{2}$ w.l.o.g. By Theorem 2, since $C_{1}\cap C_{2}⊇B\neq \emptyset$, we have $C_{2}\subseteq C_{1}$, contradicting the minimality of $C_{1}$ as desired. Hence, p(B) exists and is unique. □

### Appendix A.8

**Proof** **of** **Corollary** **2.** To prove the “if” case, consider any $B\in C_{\sigma (C)}\left(C\right)$, on one hand, B⊊C; on the other hand, by definition of $C_{\alpha }\left(\cdot \right)$ in (14), we have σ(B)>σ(C). Hence, *C* satisfies the condition in (29). To show minimality, note that the maximality of $B\in C_{\sigma (C)}\left(C\right)$ according to the definition of $C_{\alpha }\left(\cdot \right)$ in (14) indicates that

$$\begin{array}{c} \forall C^{\prime }\subseteq C:B⊊C^{\prime },\sigma \left(C^{\prime }\right)\leq \sigma \left(C\right). \end{array}$$

Hence, p(B)=C. Consider the reverse case, i.e., p(B)=C and any $\alpha^{\prime }\in R$ s.t. $B\in C_{\alpha^{\prime }}\left(V\right)$. Then

$$\begin{array}{c} \sigma \left(B\right)>\alpha^{\prime }\geq \sigma \left(C\right), \end{array}$$

where the last inequality is by Theorem 2 because $C\notin C_{\alpha^{\prime }}\left(V\right)$ as C⊋B. To show $B\in C_{\sigma (C)}\left(C\right)$, it suffices to show maximality of *B* in $C_{\sigma (C)}\left(C\right)$, i.e.,

$$\begin{array}{c} \forall C^{\prime }⊊C:C^{\prime }⊋B,\sigma \left(C^{\prime }\right)\leq \sigma \left(C\right). \end{array}$$

Suppose, on the contrary, that there exists

$$\begin{array}{c} C^{\prime }⊊C:C^{\prime }⊋B,\sigma \left(C^{\prime }\right)>\sigma \left(C\right). \end{array}$$

Then, there must also exist

$$\begin{array}{c} C^{\prime \prime }\in C_{\sigma (C)}\left(C\right):C^{\prime \prime }⊇C^{\prime }. \end{array}$$

By Theorem 2, since $C^{\prime \prime }\cap C⊇B\neq \emptyset$, we must have $C^{\prime \prime }⊊C$, contradicting the minimality of *C* by definition of parent in (29) as desired. Hence, Corollary 2 is established. □

### Appendix A.9

**Proof** **of** **Theorem** **3.** When |U|≤1, we have $C_{\alpha }\left(U\right)=\emptyset$ by the definition of $C_{\alpha }\left(\cdot \right)$ in (10), then (31) is implied by (32). Then we prove the equality in (32) by showing that for |V|≥2,

(A33) $$\begin{array}{c} \left(B_{\alpha }^{*}\left(V\right)\setminus \{\{i\}|i\in V\}\right)\subseteq C_{\alpha }\left(V\right). \end{array}$$

and

(A34) $$\begin{array}{c} C_{\alpha }\left(V\right)\setminus B_{\alpha }^{*}\left(V\right)=C_{\alpha }\left(U\right). \end{array}$$

To show (A33), consider any $B\in B_{\alpha }^{*}\left(V\right):\left|B\right|\geq 2$. By minimality of *B* in $B_{\alpha }\left(V\right)$,

fα(B)>minT⊆BT≠∅fα(T),

which implies

$$\begin{array}{c} \sigma (B)>\alpha \end{array}$$

by (23) and (24) since |B|>1. Then by Proposition 5, *B* is maximal among

(A35) $$\begin{array}{c} \{B^{\prime }\;|\;B^{\prime }\subseteq V,\sigma \left(B^{\prime }\right)>\alpha \}, \end{array}$$

which means $B\in C_{\alpha }\left(V\right)$. It remains to show (A34). First, we show that any element of the left-hand side of (A34) is on the right-hand side of (A34). Consider any element *C* of the left-hand side of (A34), i.e.,

(A36) $$\begin{array}{cc} \;\;C & \in C_{\alpha }\left(V\right),\;\mathrm{and}\; \end{array}$$

(A37) $$\begin{array}{cc} C & \notin B_{\alpha }^{*}\left(V\right). \end{array}$$

Since $C\in B_{\alpha }^{*}\left(V\right)$, we have

(A38) $$\begin{array}{c} \sigma (C)>\alpha . \end{array}$$

For any $B\in B_{\alpha }^{*}\left(V\right)$, we deduce that

(A39) $$\begin{array}{c} C⊈B. \end{array}$$

This is because,

- when |B|=1, (A39) trivially, otherwise (A36) does not hold by (10).
- when |B|>1, by (A33),
  (A40) $$\begin{array}{c} B\in C_{\alpha }\left(V\right). \end{array}$$
  Then by (A37),
  $$\begin{array}{c} C\neq B, \end{array}$$
  which further implies (A39) by the maximality of $C\in C_{\alpha }\left(V\right)$ according to (10).

By (A36), (A37), (A39) and Proposition 5,

$$\begin{array}{c} B\cap C=\emptyset . \end{array}$$

Then

$$\begin{array}{c} C\subseteq V\setminus ⋃B_{\alpha }^{*}\left(V\right), \end{array}$$

which is equivalent to C⊆U by definition of *U*, and hence by (A36),

(A41) $$\begin{array}{c} C\in C_{\alpha }\left(U\right). \end{array}$$

Next, we show that any element of the left-hand side of (A34) is in the right-hand side of (A34). Consider any element *C* of the right-hand side of (A34), i.e.,

(A42) $$\begin{array}{c} C\in C_{\alpha }\left(U\right), \end{array}$$

then by (10), we have

(A43) $$\begin{array}{c} \sigma (C)>\alpha . \end{array}$$

For any

(A44) $$\begin{array}{c} C^{\prime }\subseteq V:C^{\prime }⊋C, \end{array}$$

we deduce that

(A45) $$\begin{array}{c} \sigma (C^{\prime })\leq \alpha . \end{array}$$

This is because

- when $C^{\prime }\subseteq U$, (A45) holds by the maximality of *C* in $C_{\alpha }\left(U\right)$ indicated by (10).
- when $C^{\prime }⊈U$,
  (A46a) $$\begin{array}{cc} \; & \;\;\exists B\in B_{\alpha }^{*}\left(V\right)\;s.t. \end{array}$$
  (A46b) $$\begin{array}{cc} \; & B\cap C^{\prime }\neq \emptyset \end{array}$$
  since the way *U* is defined in (33) means any elements not in *U* must appear in subsets in $B_{\alpha }^{*}\left(V\right)$. Additionally,
  (A47) $$\begin{array}{c} C^{\prime }⊈B, \end{array}$$
  otherwise (A44) implies C⊊B, which further implies (A42) must not hold. According to Proposition 5, (A46a), (A46b) and (A47) implies (A45) holds.

(A45) implies *C* is maximal among subsets in

$$\begin{array}{c} \{C^{\prime \prime }|C^{\prime \prime }\subseteq V,\sigma \left(C^{\prime \prime }\right)>\alpha \}, \end{array}$$

which further implies

(A48) $$\begin{array}{c} C\in C_{\alpha }\left(V\right). \end{array}$$

According to (10), (A42) implies

(A49) $$\begin{array}{c} C\subseteq U. \end{array}$$

By (33), any element appeared in subsets in $B_{\alpha }^{*}\left(V\right)$ is not in *U*, then (A49) implies all the elements in *C* is not in any subset in $B_{\alpha }^{*}\left(V\right)$. Hence

(A50) $$\begin{array}{c} C\notin B_{\alpha }^{*}\left(V\right) \end{array}$$

As a result, (A48) and (A50) implies

$$\begin{array}{c} C\subseteq \left(C_{\alpha }\left(V\right)\setminus B_{\alpha }^{*}\left(V\right)\right). \end{array}$$

Hence, (A34) is established. The combination of (A33) and (A34) establish (32) in Theorem 3. Hence, the equality in the recurrence relation holds. By Proposition 4, the recursive procedure ends in finite steps. Hence, Theorem 3 is valid for calculating $C_{\alpha }\left(V\right)$ recursively. □

### Appendix A.10

**Proof** **of** **Proposition** **3.** When |V|≥2, the feasible domain of (18) is non-empty, then its solution set $B_{\alpha }\left(V\right)$ is non-empty, and hence $B_{\alpha }^{*}\left(V\right)$ is non-empty, i.e., (34) holds. □

### Appendix A.11

**Proof** **of** **Proposition** **4.** When |V|=1, it corresponds to the base case in (31), hence only need 1 recursive step to calculate $C_{\alpha }\left(V\right)$. When |V|≥2, for $N^{\prime }\in N$, suppose $U_{i},U_{2},\cdots ,U_{N^{\prime }-1}$ is the sequence of the ground set for computing $C_{\alpha }\left(\cdot \right)$ used in the recursive steps that corresponds to (32), and $U_{N^{\prime }}$ is the ground set for computing $C_{\alpha }\left(\cdot \right)$ by the base case (31). Then we know $U_{1}=V$, $|U_{N^{\prime }}\left|\in \{0,1\right\}$ and $N^{\prime }$ is the number of recursive steps. Proposition 3 implies that $|U_{i+1}\left|\leq \right|U_{i}|-1$ for $i=1,2,\cdots ,N^{\prime }-1$. Hence $N^{\prime }\leq \left|V\right|$. Hence, Proposition 4 holds. □

### Appendix A.12

**Proof** **of** **Proposition** **5.** Suppose on the contrary that there exists C⊆V s.t. σ(C)>α, B∩C=∅ and C⊈B. Consider an inclusion-wise maximal *C*. By Lemma 1 we have

$$\begin{array}{cc} \sigma (C\cup B) & \geq \min\{\sigma (C),\sigma (B)\}\\ \; & >\alpha , \end{array}$$

and so, by maximality of *C* we have C⊋B. Then we have

fα(C)<(a)minT⊊C:T≠∅fα(T)≤(b)fα(B),

where (a) is by (23) and (24) in Proposition 2, and (b) is by C⊋B as obtained above with the assumption. However, this contradicts the optimality of $B\in B_{\alpha }^{*}\left(V\right)$. □

### Appendix A.13

**Proof** **of** **Proposition** **6.** 

(A51) $$\begin{array}{cc} B_{\alpha }^{*}\left(V\right)\; & \overset{\left(a\right)}{=}\mathrm{minimal}\;\underset{t\in T_{\alpha }^{*}\left(V\right)}{⋃}\underset{B\subseteq C:t\in B}{arg min}f_{\alpha }\left(B\right) \end{array}$$

(A52) $$\begin{array}{cc} \; & \overset{\left(b\right)}{=}\mathrm{minimal}\;\underset{t\in T_{\alpha }^{*}\left(V\right)}{⋃}\left(\mathrm{minimal}\;\underset{B\subseteq C:t\in B}{arg min}f_{\alpha }\left(B\right)\right) \end{array}$$

(A53) $$\begin{array}{cc} \; & \;\overset{\left(c\right)}{=}\mathrm{minimal}\;\underset{t\in T_{\alpha }^{*}\left(V\right)}{⋃}B_{\alpha }^{\left(t\right)}\left(V\right), \end{array}$$

where (a) is by definition of $B_{\alpha }^{*}\left(V\right)$, (20) and (37); (b) is by the fact that for a $t\in T_{\alpha }^{*}\left(V\right)$ and any $A\in {arg min}_{B\subseteq C:t\in B}f_{\alpha }\left(B\right)$, if *A* is not minimal, then *A* is not in the right-hand side due to the outer minimal applied to the union; and (c) is by definition of $B_{\alpha }^{\left(t\right)}\left(V\right)$. □

### Appendix A.14

**Proof** **of** **Proposition** **7.** When |V|≤1, the base case (31) applies, and it takes O(1) time. When |V|≥2, the recursive case (32) applies. Consider the recursive step with ground set *V* in Theorem 3:

- For a t∈V, calculating $B_{\alpha }^{\left(t\right)}\left(V\right)$ for all α∈R takes MNB(|V|) time by the minimum norm base algorithm, hence calculating $B_{\alpha }^{\left(t\right)}\left(V\right)$ for all t∈V,α∈R takes ${O(|V|}^{2}\mathrm{MNB}(|V|))$ time;
- Calculating $T_{\alpha }^{*}\left(V\right)$ for an α∈R takes O(|V|) time, hence calculating $T_{\alpha }^{*}\left(V\right)$ for all α∈R takes $O(|V{|}^{3})$ time, since there can be a number O(|V|) of α values where we need to conduct calculation;

Hence it takes ${O(|V|}^{2}\mathrm{MNB}(|V|))$ time for this recursive step. According to Proposition 4, the recursive procedure ends with at most |V| recursive steps, hence it takes ${O(|V|}^{3}\mathrm{MNB}(|V|))$ time to calculate ${⋃}_{\alpha \in R}C_{\alpha }\left(V\right)$. □

### Appendix A.15. Calculation of Strong Communities in Figure 3b

As an example, we show how to obtain the dendrogram in Figure 3b and then obtain the strong communities below.

Following the recursive procedure in Theorem 3, to solve $C_{\alpha }\left(V\right)$, first seek to solve $B_{\alpha }^{*}\left(V\right)$, then for each critical α value at turning point, solve for $C_{\alpha }\left(U\right)$ with similar procedure, where *U* is the complement set of V given by (33). Hence, the procedure to calculate $B_{\alpha }^{*}\left(V\right)$ is representative, which can be done according to Proposition 6.

When j=1 is the sink node,

$$\begin{array}{cc} f_{\alpha }\left(\left\{1\right\}\right) & =\alpha \cdot |\left(\{1\}\right)|-g(\{1\})=\alpha ,\\ f_{\alpha }\left(\left\{1,2\right\}\right) & =\alpha \cdot |\left(\{1,2\}\right)|-g(\{1,2\})=2\alpha -4,\\ f_{\alpha }\left(\left\{1,2,3\right\}\right) & =3\alpha -5,\\ f_{\alpha }\left(\left\{1,2,3,4\right\}\right) & =4\alpha -8, \end{array}$$

and $f_{\alpha }\left(\left\{1,2,4\right\}\right),f_{\alpha }\left(\left\{1,3\right\}\right),f_{\alpha }\left(\left\{1,3,4\right\}\right),f_{\alpha }\left(\left\{1,4\right\}\right)$ can be written out similarly and we omit them here. Then drawing $f_{\alpha }$ against α, the lowest curve formed is ${\hat{f}}_{\alpha }^{\left(1\right)}\left(V\right)$, and the corresponding solution $B_{\alpha }^{\left(1\right)}\left(V\right)$ can be obtained.

$$\begin{array}{c} {\hat{f}}_{\alpha }^{\left(1\right)}\left(V\right)=\left\{\begin{array}{cc} 4\alpha -8, & \alpha <2,\\ 2\alpha -4, & 2\leq \alpha <4,\\ \alpha , & \alpha \geq 4, \end{array}\right. \end{array}$$

and correspondingly,

$$\begin{array}{c} B_{\alpha }^{\left(1\right)}\left(V\right)=\left\{\begin{array}{cc} \left\{\{1,2,3,4\}\right\}, & \alpha <2,\\ \left\{\{1,2\}\right\}, & 2\leq \alpha <4,\\ \left\{\{1\}\right\}, & \alpha \geq 4, \end{array}\right. \end{array}$$

Then, for all j∈V, calculate ${\hat{f}}_{\alpha }^{\left(j\right)}\left(V\right)$ and $B_{\alpha }^{\left(j\right)}\left(V\right)$, and finally obtain $T_{\alpha }^{*}\left(V\right)$ and $B_{\alpha }^{*}\left(V\right)$.

The result is

$$\begin{array}{c} T_{\alpha }^{*}\left(V\right)=\left\{\begin{array}{cc} \{1,2,3,4\}, & \alpha <2,\\ \{1,2\}, & 2\leq \alpha <4,\\ \{1,2,3,4\}, & \alpha \geq 4, \end{array}\right. \end{array}$$

$$\begin{array}{c} B_{\alpha }^{*}\left(V\right)=\left\{\begin{array}{cc} \left\{\{1,2,3,4\}\right\}, & \alpha <2,\\ \left\{\{1,2\}\right\}, & 2\leq \alpha <4,\\ \left\{\{1\},\{2\},\{3\},\{4\}\right\}, & \alpha \geq 4, \end{array}\right. \end{array}$$

and

$$\begin{array}{c} B_{\alpha }^{*}\left(V\right)\setminus \{\{i\}i\in V\}=\left\{\begin{array}{cc} \left\{\{1,2,3,4\}\right\}, & \alpha <2,\\ \left\{\{1,2\}\right\}, & 2\leq \alpha <4,\\ \emptyset , & \alpha \geq 4, \end{array}\right. \end{array}$$

When α<2 or α≥4, the *U* in (33) satisfies (31), hence the base case applies, no new strong subset is found and the recursion finishes.

For 2≤α<4, we need to continue to calculate $C_{\alpha }\left(U\right)$ with U={3,4}. With a similar calculation procedure, we can obtain for 2≤α<4,

$$\begin{array}{c} B_{\alpha }^{*}\left(U\right)=\left\{\begin{array}{cc} \left\{\{3,4\}\right\}, & 2\leq \alpha <3,\\ \left\{\{3\},\{4\}\right\}, & 3\leq \alpha <4, \end{array}\right. \end{array}$$

and

$$\begin{array}{c} B_{\alpha }^{*}\left(U\right)\setminus \{\{i\}|i\in U\}=\left\{\begin{array}{cc} \left\{\{3,4\}\right\}, & 2\leq \alpha <3,\\ \emptyset , & 3\leq \alpha <4. \end{array}\right. \end{array}$$

Then the remaining recursions to be done satisfy the base case in (31). Hence all the strong communities are obtained. The dendrogram of the strong communities is shown in Figure 3b.
